# *In Silico* Approach towards Designing Virtual Oligopeptides for HRSV

**DOI:** 10.1155/2014/613293

**Published:** 2014-11-27

**Authors:** Ruchi Jain, Shanmughavel Piramanayagam

**Affiliations:** DBT-Bioinformatics Centre, Computational Biology Lab, Department of Bioinformatics, Bharathiar University, Coimbatore, Tamil Nadu 641 046, India

## Abstract

HRSV (human respiratory syncytial virus) is a serious cause of lower respiratory tract illness in infants and young children. Designing inhibitors from the proteins involved in virus replication and infection process provides target for new therapeutic treatments. In the present study, *in silico* docking was performed using motavizumab as a template to design motavizumab derived oligopeptides for developing novel anti-HRSV agents. Additional simulations were conducted to study the conformational propensities of the oligopeptides and confirmed the hypothesis that the designed oligopeptide is highly flexible and capable of assuming stable confirmation. Our study demonstrated the best specific interaction of GEKKLVEAPKS oligopeptide for glycoprotein strain A among various screened oligopeptides. Encouraged by the results, we expect that the proposed scheme will provide rational choices for antibody reengineering which is useful for systematically identifying the possible ways to improve efficacy of existing antibody drugs.

## 1. Introduction

HRSV, a* Pneumovirus* in the family Paramyxoviridae, is the single most important cause of serious lower respiratory tract illnesses such as bronchiolitis and pneumonia in infants and young children [1–3]. RSV is increasingly recognized as an important nosocomial pathogen causing morbidity in immune compromised patients [4]. Estimated number of individuals infected from lower respiratory tract infections in 2005 accounted for more than 30 million, each year resulting in nearly 3 million hospitalizations under 5 years of age, which makes it the most common cause of hospitalization in children [5]. Nonspecific antiviral, that is, Ribavirin, hampers virus transcription; however, many symptoms are grouped and its viability represents the need for more potent and safe therapeutics to treat HRSV infection [6, 7]. Humanized monoclonal antibody called palivizumab is used to prevent HRSV-induced respiratory tract disease in high-risk infants [8, 9], while motavizumab is an affinity optimized monoclonal antibody developed from palivizumab and has been assessed clinically [9–11]. In later research, both palivizumab and motavizumab failed in virus attachment and were incapable of interacting with the target cell membrane. Besides this, Food and Drug Administration's (FDA's) Antiviral Drugs Advisory Committee panel voted not to suggest motavizumab for licensure, raising concerns about hypersensitivity and skin rash occurring within two days of dosing. MedImmune withdrew its requisition for licensure of motavizumab and affirmed that the product will not be further developed for immunoprophylaxis of serious HRSV infection [12].

Endeavours to develop an HRSV vaccine have so far floundered owing to issues with no long term cure and potency. Next generation antibodies in which antibody structural modifications are adopted are an exertion to enhance immunoprophylactic therapy and few antibodies are being developed and as of now advancing through clinical development. Present study has implemented* in silico* methodologies to design oligopeptide derived from the interacting residues of surface proteins and the antibody. Surface proteins such as glycoprotein (involved in host cell attachment), F protein (directs viral penetration by membrane fusion and also mediates fusion of infected cells with their neighbours to form syncytia), matrix protein (important in virion morphogenesis), and small hydrophobic protein (involved in infection and replication) were targeted for the present study since these viruses are involved in fusion and replication and infection processes. Binding affinity was cross-checked further by studying interaction from motavizumab derived interacting residues (hereafter referred to as original oligopeptides) and designed oligopeptides (random shuffling of original oligopeptides); on the other hand, simulation studies were performed to ensure the stability of the designed oligopeptides adding the peptide property calculations to validate it, thus proving designed oligopeptides mimicking the role of motavizumab in a better way.

## 2. Materials and Methods

### 2.1. Molecular Interaction of Antibody with Viral Proteins

The structure of fusion protein was retrieved from Protein Data Bank (PDB ID: 1G2C) whereas structure of glycoproteins A and B, matrix protein, and small hydrophobic protein was modeled using I-TASSER [13] and has been validated by SAVES (structural analysis and verification server). The structure of motavizumab (PDB ID: 4JLR) was subjected to dock against the surface protein structures, that is, glycoproteins A and B, fusion, matrix, and small hydrophobic protein through BioLuminate module; it integrates PIPER, a protein-protein docking module by Schrödinger software suite. Energies of billions of docked conformations can be evaluated on a grid using fast Fourier transform (FFT) correlation approach embedded in PIPER. The retained structures were clustered using the pairwise root mean square deviation (RMSD) as the distance measure with a fixed or variable clustering radius through PIPER. Interaction of the antibody and surface proteins was then analysed using PDBsum generate [14]. Interacting residues were taken and arranged according to their occurrence in the main sequence and oligopeptides were designed. To get insights into the binding affinity of the designed oligopeptide, amino acid positions within the oligopeptide were altered randomly and possible oligopeptides were created.

### 2.2. Modeling of Interacting Residues of Antibodies

Structure modeling of the designed oligopeptides was done with PEPstr [15] which predicts tertiary structures of the interacting residues; it uses predictions from PSIPRED and beta-turns (*β*-turns) for regular secondary structure information and *β*-turns information, respectively. Backbone-dependent rotamer library was used for placing side-chain angles with energy minimization and molecular dynamic simulations by using Amber version 6.

### 2.3. Analysis of Oligopeptides and Their Residues Properties

Properties of predicted oligopeptide structure were calculated using peptide-property-calculator [16] which includes isoelectric point, log⁡*P* (octanol-water partition coefficient), log⁡*S* (water solubility), and hydropathy plots and which allows for the visualization of hydrophobicity over the length of a peptide sequence. Hydrophobic and hydrophilic properties of the amino acids are plotted on hydropathy scale [17].

### 2.4. Conformation and Potential Energy Prediction

Amino acid sequence was shuffled and number of variants was produced. MD simulation of the variants produced was performed using Macromodel Version 9.0 from Schrödinger suite. The OPLS_2005 force field was used for the energy calculation. Constant temperature was 300 K and in the integration step 1.0 fs was given. MD simulation with position restraints was carried out for a period of 1 ns in order to allow the accommodation of the water molecules in the system.

### 2.5. Interaction Studies of Proteins and Oligopeptides

Interaction studies were carried out using ZDOCK server [18]. It searches all rotational and translational space for the ligand protein relative to the receptor protein. Original and designed oligopeptides of each protein were docked taking motavizumab structure from PDB (PDB ID: 4JLR) as a target protein, only heavy chain and light chain were considered for docking, and *Z*-score was calculated on the basis of pairwise shape complementarity function (total number of receptor-ligand atom pairs within a distance cutoff minus a clash penalty), desolvation energy (free energy change of breaking two protein atom-water contacts and forming a protein atom-protein atom contact and a water-water contact), and electrostatic energy (function of the electrical potential generated by the receptor and the partial charges of ligand atoms).

## 3. Results

**Figure 1:**
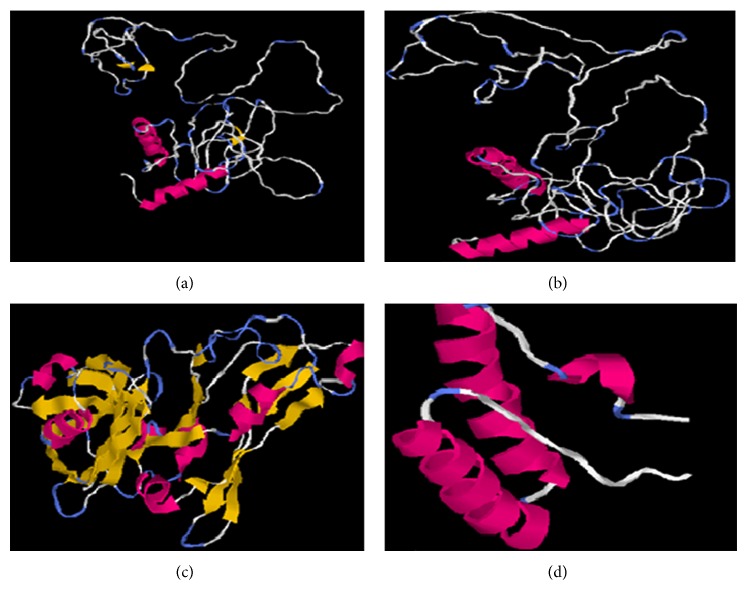
Modeled structures of (a) glycoprotein A, (b) glycoprotein B, (c) matrix protein, and (d) small hydrophobic protein.

**Figure 2:**
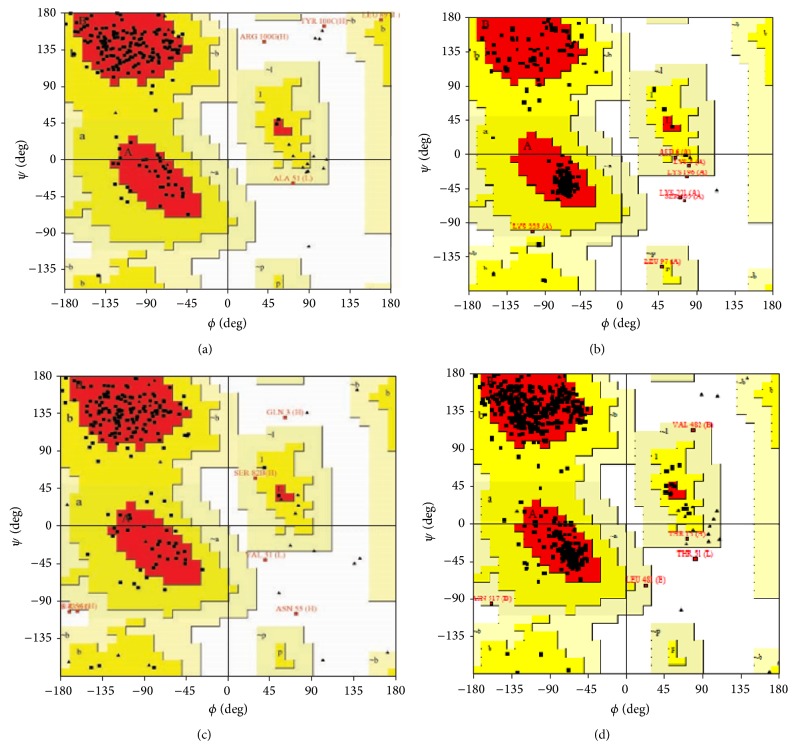
Ramachandran plot of (a) glycoprotein A, (b) glycoprotein B, (c) matrix protein, and (d) small hydrophobic protein. 89.7% of the residues from glycoprotein A, 90.3% of the residues from glycoprotein B, 79.3% of the residues of matrix protein, and 89.9% of the residues from small hydrophobic protein present in most favoured regions.

**Figure 3:**
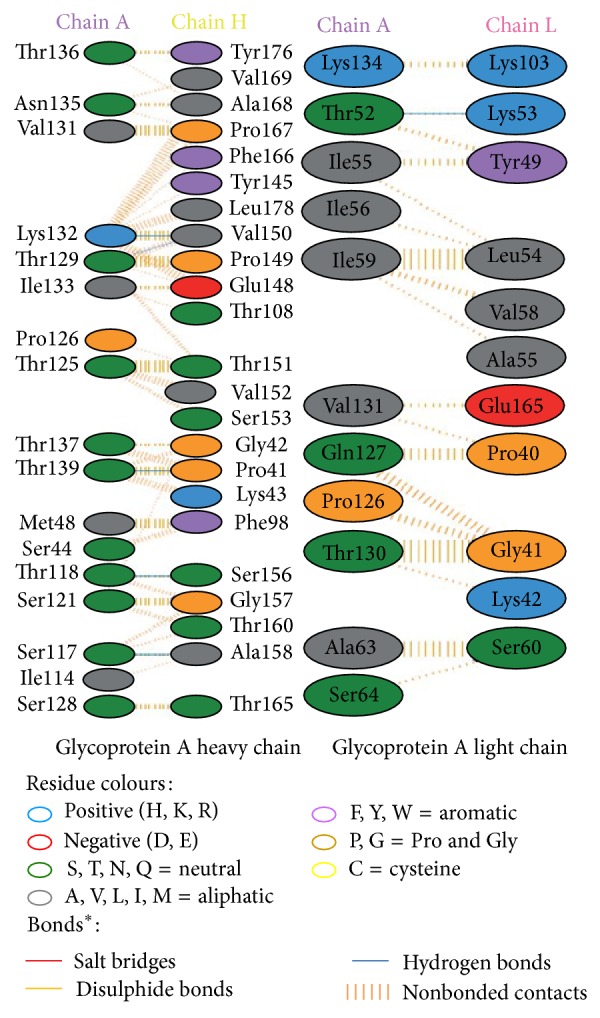
Interaction of glycoprotein A and motavizumab showing interaction of residues with heavy and light chain through various bonds^*^.

**Figure 4:**
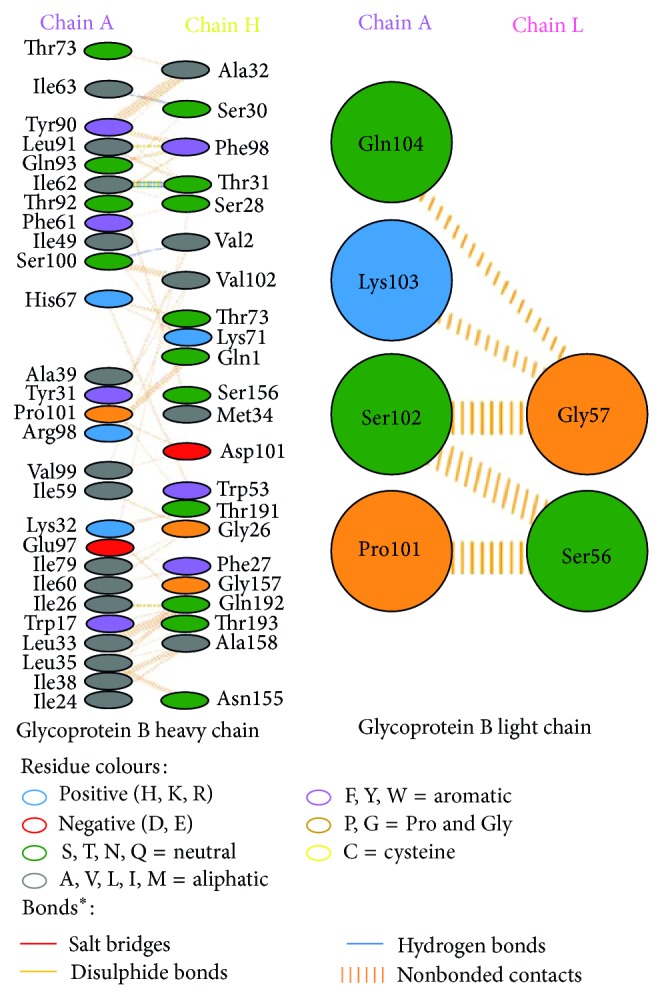
Interaction of glycoprotein B and motavizumab showing interaction of residues with heavy and light chain through various bonds^*^.

**Figure 5:**
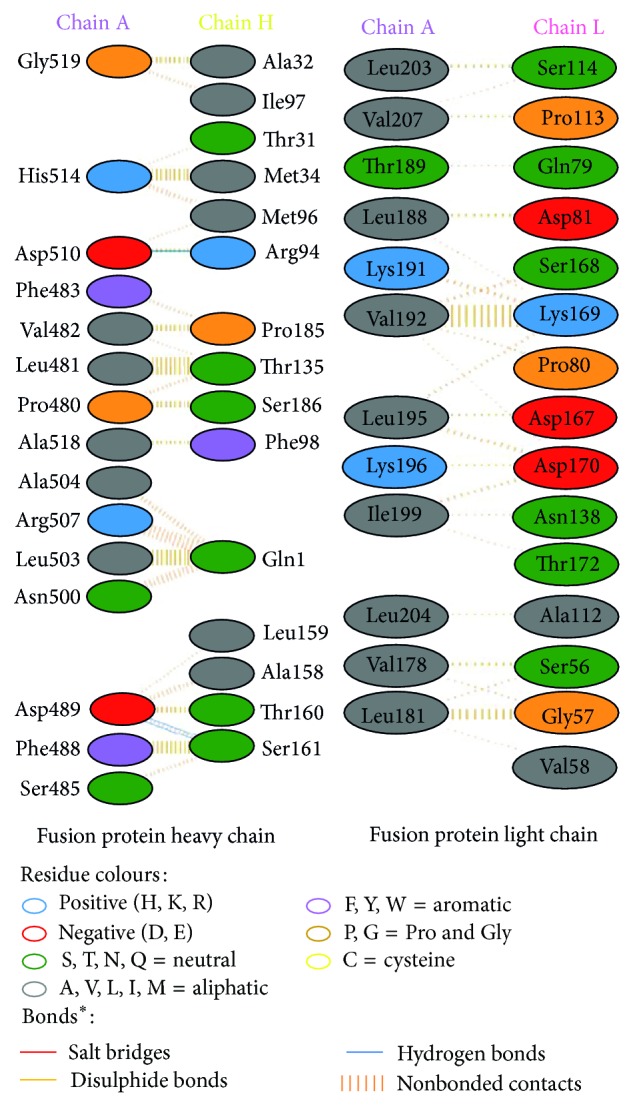
Interaction of fusion protein and motavizumab showing interaction of residues with heavy and light chain through various bonds^*^.

**Figure 6:**
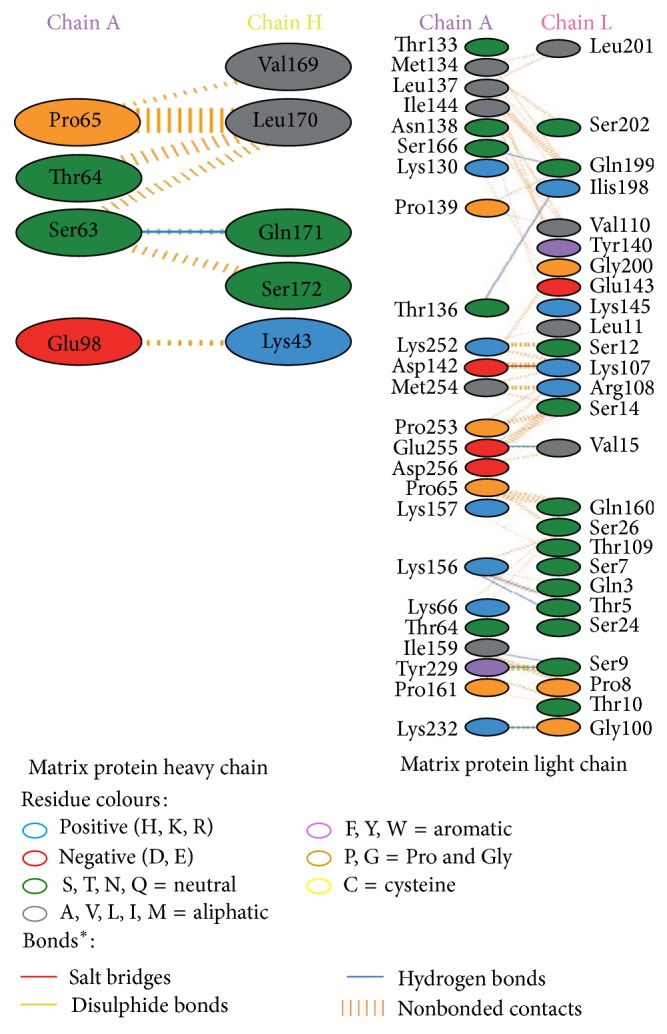
Interaction of matrix protein and motavizumab showing interaction of residues with heavy and light chain through various bonds^*^.

**Figure 7:**
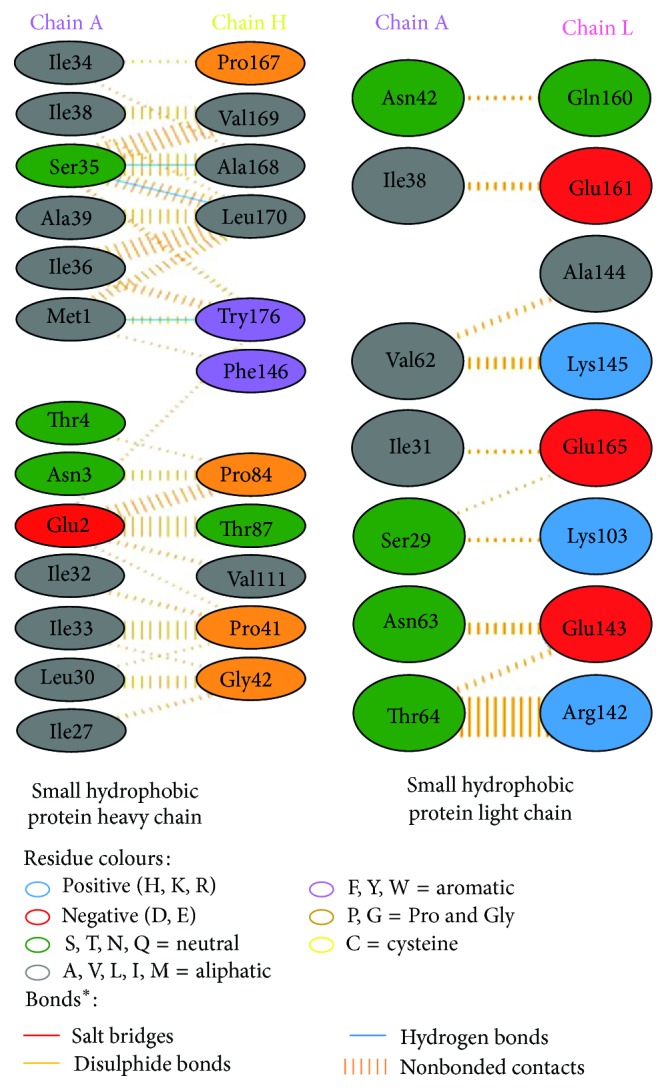
Interaction of small hydrophobic protein and motavizumab showing interaction of residues with heavy and light chain through various bonds^*^.

Modeled structures of glycoproteins A and B, matrix protein, and small hydrophobic proteins were represented in Figure 1. All modeled structures were validated through Structural Analysis and Verification Server (SAVES) [19]. Ramachandran plot showed that 90.3% of the residues from glycoprotein A, 83.4% of the residues from glycoprotein B, 86.5% of the residues of matrix protein, and 88.3% of the residues from small hydrophobic protein were present in most favoured regions (Figure 2). Motavizumab was subjected to interact with all surface proteins. Heavy chain (H chain) of motavizumab interacted with glycoprotein A through five hydrogen bonds and nonbond interactions while light chain (L chain) interacted with one hydrogen bond and nonbond interactions (Figure 3). H chain of motavizumab bound with glycoprotein B by four hydrogen bonds and nonbond interactions while no hydrogen bond was found in the interaction with L chain showing all nonbond interactions in Figure 4. H chain of motavizumab formed three hydrogen bonds and nonbonded interaction whereas no hydrogen bond was found when motavizumab interacted with L chain of fusion protein (Figure 5). H chain of motavizumab interacted with matrix protein and formed one hydrogen bond and nonbond interactions whereas L chain of motavizumab interacted with matrix protein and formed eight hydrogen bonds, one salt bridge, and nonbond interaction (Figure 6). Finally H chain of motavizumab bound with small hydrophobic protein with three hydrogen bonds and other nonbond interactions while no hydrogen bond was formed when it interacted with L chain of motavizumab; only nonbonded interactions were seen (Figure 7).

**Figure 8:**
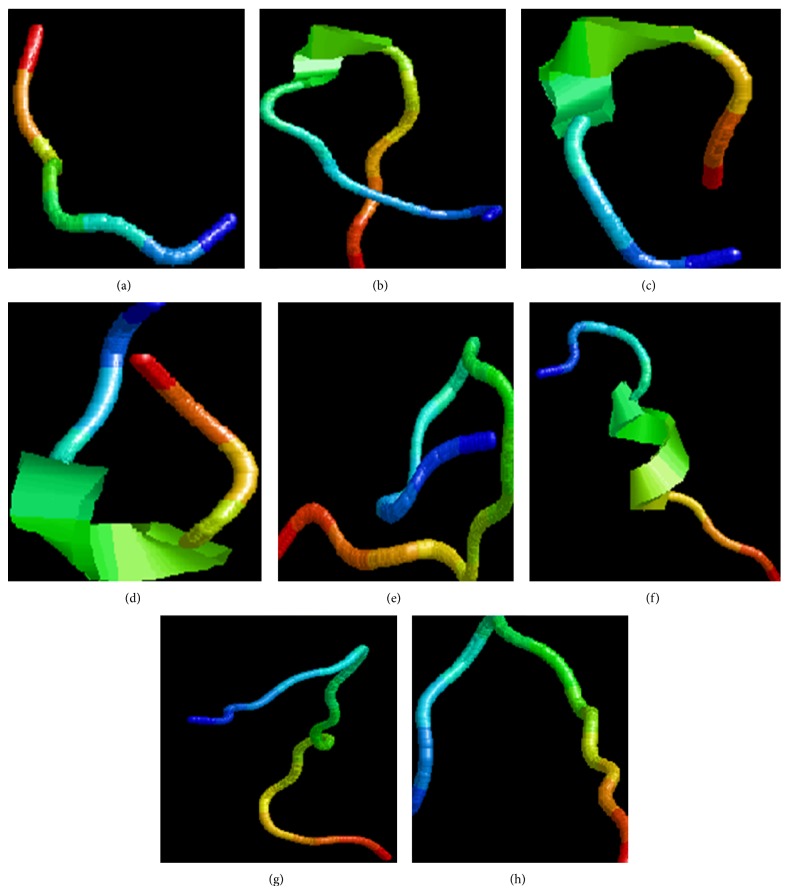
Ribbon representation of the modeled oligopeptides. (a) KREAKQEE, (b) PGKFYVSSGATTFPAVYL, (c) PGKYKLAVSEK, (d) PGPTVFPAVLY, (e) PSTLSVSSGTVYEKHEGLS, (f) QTAMRMIFTALTSPS, (g) QVGFSSTAMWNKTFDVSGATQ, and (h) SGVQPDAPSNDSKDT.

Interacting residues of motavizumab with all proteins were retrieved and arranged in sequential order (Table 1). 3D structure of oligopeptide modeled from interacting residues was represented in Figure 8. Properties such as isoelectric point, log⁡*P* (octanol-water partition coefficient), log⁡*S* (pH dependent aqueous solubility), and water solubility of oligopeptides were calculated, which shows that PGKYKLAVSEK (derived from motavizumab and glycoprotein A interaction), SGVQPDAPSNDSKDT (derived from motavizumab and fusion protein interaction), PSTLSVSSGTVYEKHEGLS (derived from motavizumab and matrix protein interaction), and KREAKQEE (derived from motavizumab and small hydrophobic protein interaction) have good water solubility while all others show poor water solubility (Table 2). These oligopeptides (original oligopeptide) were further targeted and residues were shuffled randomly and possible oligopeptides were designed (designed oligopeptides) (Table 3).

**Figure 9:**
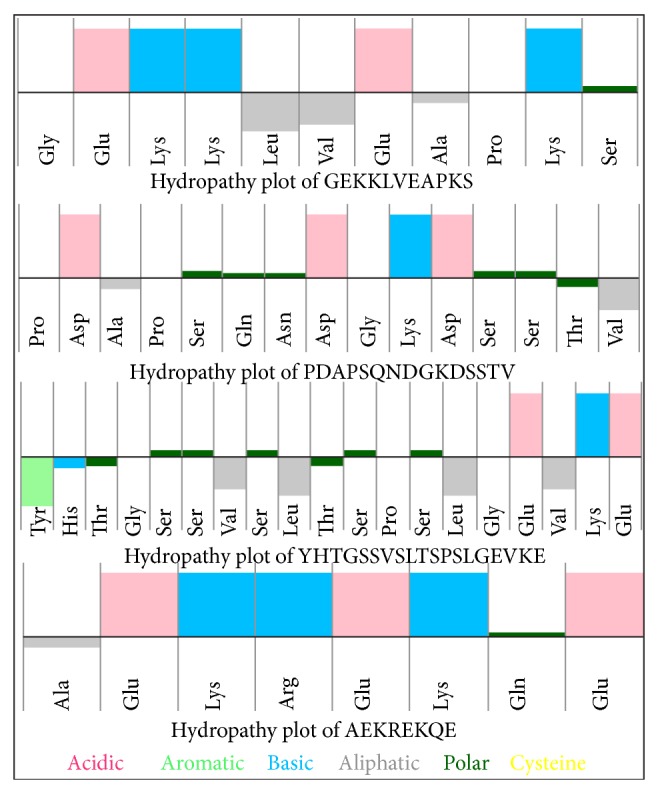
Hydropathy plot of GEKKLVEAPKS, PDAPSQNDGKDSSTV, YHTGSSVSLTSPSLGEVKE, and AEKREKQE. Composition of amino acid in a peptide is represented in coloured format showing top region as hydrophilic and bottom region as hydrophobic.

Simulation studies were performed for all original and designed oligopeptides using Macromodel Schrodinger suite for 1 ns and scatter plot was plotted. We seek to optimize the propensity of the oligopeptides to assume the stable conformation and thus by considering the potential energy for all original and designed oligopeptides a combined graph was plotted for each oligopeptide. The graph shows that, among all designed oligopeptides, SSGAYVTTFPKFAVYGLP from PGKFYVSSGATTFPAVYL and GEKKLVEAPKS from PGKYKLAVSEK (derived from motavizumab and glycoprotein A interaction), NKATATGVQGMTDFGSVSSQW from QVGFSSTAMWNKTFDVSGATQ (derived from motavizumab and glycoprotein B interaction), MIFQLTTSSAPARTM from QTAMRMIFTALTSPS and PDAPSQNDGKDSSTV from SGVQPDAPSNDSKDT (derived from motavizumab and fusion protein interaction), YHTGSSVSLTSPSLGEVKE from PSTLSVSSGTVYEKHEGLS (derived from motavizumab and matrix protein interaction), and PPVAYLTPFVG from PGPTVFPAVLY and AEKREKQE from KREAKQEE (derived from motavizumab and small hydrophobic protein interaction) were the most stable in terms of potential energy as represented in supplementary figure (see Supplementary Material available online at http://dx.doi.org/10.1155/2014/613293). Among them, the oligopeptides which are the most stable and have good water solubility were GEKKLVEAPKS, PDAPSQNDGKDSSTV, YHTGSSVSLTSPSLGEVKE, and AEKREKQE; hydropathy plot in Figure 9 represents top region as hydrophilic and bottom region as hydrophobic [20].

**Figure 10:**
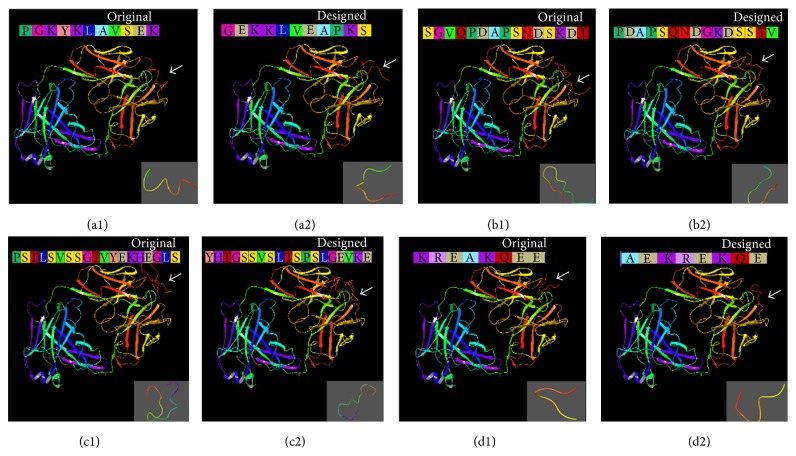
Interaction of antibody with original and designed oligopeptides; arrow represents binding site of oligopeptide with antibody, as shown in the lower right side of each figure. (a1) represents original PGKYKLAVSEK, (a2) represents designed oligopeptide GEKKLVEAPKS, (b1) represents original SGVQPDAPSNDSKDT, (b2) represents designed oligopeptide PDAPSQNDGKDSSTV, (c1) represents original PSTLSVSSGTVYEKHEGLS, (c2) represents designed oligopeptide YHTGSSVSLTSPSLGEVKE, (d1) represents original KREAKQEE, and (d2) designed oligopeptide AEKREKQE.

For further screening among these designed oligopeptides, molecular docking procedure was performed by ZDOCK server for original oligopeptide and designed oligopeptide to check their affinity towards respective proteins (Figure 10) which showed that *Z*-score of PGKYKLAVSEK is 964.281 and designed oligopeptide (GEKKLVEAPKS) *Z*-score is 994.764, whereas the *Z*-score of SGVQPDAPSNDSKDT is 937.834 and designed oligopeptide (PDAPSQNDGKDSSTV) *Z*-score is 632.903, similarly PSTLSVSSGTVYEKHEGLS *Z*-score is 868.628 and designed oligopeptide (YHTGSSVSLTSPSLGEVKE) *Z*-score is 654.164, and finally KREAKQEE *Z*-score is 576.178 and designed oligopeptide (AEKREKQE) *Z*-score is 572.8880. Based on the binding efficacy and peptide properties of the designed oligopeptide from PGKYKLAVSEK, GEKKLVEAPKS can be considered for further studies.

## 4. Discussion

Antibodies can neutralize HRSV either by binding the virus surface and preventing its ability to interact with cellular receptors or by binding after virion attachment and blocking the subsequent steps involved with virus entry. Efficacy issues of motavizumab have been demonstrated and its application needs further improvement. Understanding molecular basis for designing and synthesizing new antibody is necessary for better treatment. For the same reason, understanding structural rules governing antigen-antibody interactions of a virus is also necessary [21].* In silico* approaches were used in present study where computational docking was implemented predicting docked complex of viral surface proteins and motavizumab through which interacting residues were obtained. Knowing which residues bind to the virus protein can be beneficial in antibody engineering. These residues were found to be present in CDRs (complementary determining regions) and outside the CDRs; among them, the residues that fall outside of the traditionally defined CDRs are at least as important to antigen binding as residues within the CDRs, and, in some cases, they are even more important energetically; hence we have considered all interacting residues irrespective of their occurrence [22].

Moreover, low solubility of therapeutic antibodies is more difficult in formulations and may lead to poor biodistribution, undesirable pharmacokinetics behavior, and immunogenicity [23]; for the same purpose, oligopeptides which were having good water solubility were taken in consideration; moreover, the intrinsic properties of proteins such as size, hydrophobicity, lipophilicity, and isoelectric point play important roles in absorption of antibody [23–28] which favors our findings. ZDOCK scores based on knowledge-based statistical potentials have additionally demonstrated that a modest increase in affinity through docking of GEKKLVEAPKS, a designed oligopeptide from PGKYKLAVSEK, can enhance the functional properties of motavizumab for therapeutic targeting to the HRSV cause. The use of these* in silico* techniques may provide a valuable addition to conventional experimental methods in developing improved antibodies for the treatment of HRSV cause as well as other endemic diseases.

## 5. Conclusion

Strategies for optimizing and improving antibody properties are highly desirable, either to increase their efficacy or to alter their binding specificity. Understanding sequence-structure relationships in antibodies and advances in computational methods has enabled progress that can assist in redesigning antibodies for higher affinity or other desired modifications. Using experimentally determined structure, we have performed interaction studies of antibody and protein followed by redesigning the oligopeptides derived from interacting residues, thus improving binding affinity, specificity, and other properties such as solubility. However, more validation studies are desired to know the real efficacy of the oligopeptides as an anti-HRSV drug in the future. We have generated testable hypothesis which can help to interpret and guide* in vivo *and* in vitro *experiments.

## Supplementary Material

Designed oligopeptides are differentially coloured compared with the model oligopeptide and represented at the right side of the graph which helps in identifying the most stable oligopeptide in terms of their potential energy.

## Figures and Tables

**Table 1:** Molecular interaction of proteins with motavizumab.

Protein residues	Ab residues	Chain name	One-letter code	Peptide
Glycoprotein A				
Thr136, Asn135, Val131, Lys132, Thr129, Ile133, Pro126, Thr125, Thr137, Thr139, Met48, Ser44, Thr118, Ser121, Ser117, Ile114, Ser128	Tyr176, Val169, Ala168, Pro167, Phe166, Tyr145, Leu178, Val150, Ser153, Gly42, Pro41, Lys43, Phe98, Ser156, Gly57, Thr160, Ala158, Thr165	H	P, G, K, F, Y, V, S, S, G, A, T, T, F, P, A, V, Y, L	PGKFYVSSGATTFPAVYL
Lys134, Thr52, Ile55, Ile56, Ile59, Val131, Gln127, Pro126, Thr130, Ala63, Ser64	Lys103, Lys53, Tyr49, Leu54, Val58, Ala55, Glu65, Pro40, Gly41, Lys42, Ser60	L	P, G, K, Y, K, L, A, V, S, E, K	PGKYKLAVSEK

Glycoprotein B				
Thr73, Ile63, Tyr90, Leu91, Gln93, Ile62, Thr92, Phe61, Ile49, Ser100, His67, Ala39, Tyr31, Pro101, Arg98, Val99, Ile59, Lys32, Glu97, Ile79, Ile60, Ile26, Trp17, Leu33, Leu35, Ile38, Ile24	Ala32, Ser30, Phe98, Thr31, Ser28, Val2, Val102, Thr73, Lys71, Gln1, Ser156, Met34, Asp101, Trp53, Thr191, Gly26, Phe27, Gly157, Gln192, Ala158, Asn55	H	Q, V, G, F, S, S, T, A, M, W, N, K, T, F, D, V, S, G, A, T, Q	QVGFSSTAMWNKTFDVSGATQ
Gln104, Lys103, Ser102, Pro101	Gly57, Ser56	L	S, G	SG

Fusion protein				
Leu203, Val207, Thr189, Leu188, Lys191, Val192, Leu195, Lys196, Ile199, Leu204, Val178, Leu181	Ser114, Pro113, Gln79, Asp81, Ser168, Lys169, Pro80, Asp167, Asp170, Asn138, Thr172, Ala112, Ser56, Gly57, Val58	H	S, G, V, Q, P, D, A, P, S, N, D, S, K, D, T	SGVQPDAPSNDSKDT
Gly519, His514, Asp510, Phe483, Val482, Leu481, Pro480, Ala518, Arg507, Leu503, Asn500, Asp489, Phe488, Ser485	Ala32, Ile97, Thr31, Met34, Met96, Arg94, Pro185, Thr135, Ser186, Phe98, Gln1, Leu159, Ala158, Thr160, Ser161	L	Q, T, A, M, R, M, I, F, T, A, L, T, S, P, S	QTAMRMIFTALTSPS

Matrix protein				
Pro65, Thr64, Ser63, Glu98	Val169, Leu170, Gln171, Ser172, Lys43	H	K, V, L, Q, S	KVLQS
Thr133, Met134, Leu137, Ile144, Asn138, Ser166, Lys130, Pro139, Thr136, Lys252, Asp142, Met254, Pro253, Glu255, Asp256, Pro65, Lys157, Lys156, Lys66, Thr64, Ile159, Tyr229, Pro161, Lys232	Leu201, Ser202, Gln199, His198, Val110, Tyr140, Gly200, Glu143, Lys145, Leu11, Ser14, Val15, Gln160, Ser26, Thr109, Ser24, Ser9, Pro8, Thr10, Gly100	L	P, S, T, L, S, V, S, S, G, T, V, Y, E, K, H, E, G, L, S	PSTLSVSSGTVYEKHEGLS

Small hydrophobic protein				
Ile34, Ile38, Ser35, Ala39, Ile36, Met1, Thr4, Asn3, Glu2, Ile32, Ile33, Leu30, Ile27	Pro167, Val169, Ala168, Leu170, Tyr176, Phe146, Pro84, Thr87, Val111, Pro41, Gly42	H	P, G, P, T, V, F, P, A, V, L, Y	PGPTVFPAVLY
Asn42, Ile38, Val62, Ile31, Ser29, Asn63, Thr64	Gln160, Glu161, Ala144, Lys145, Glu165, Lys103, Glu143, Arg142	L	K, R, E, A, K, Q, E, E	KREAKQEE

Interaction of motavizumab with all proteins resulted in interacting residues from heavy and light chain. These residues were combined and arranged in sequential order and thus peptides were formed.

**Table 2:** Interacting oligopeptides from proteins and their properties.

Peptide	Average log⁡*P*	Average log⁡*S*	Water solubility	Isoelectric point
PGKYKLAVSEK	−2.69	−4.82	Good	pH 10.05
PGKFYVSSGATTFPAVYL	−0.40	−6.28	Poor	pH 9.52
QVGFSSTAMWNKTFDVSGATQ	−2.47	−4.75	Poor	pH 6.75
QTAMRMIFTALTSPS	−2.50	−4.49	Poor	pH 11.04
SGVQPDAPSNDSKDT	−12.45 (+−6.32)	−1.82	Good	pH 3.59
PSTLSVSSGTVYEKHEGLS	−1.92	−3.95	Good	pH 5.3
KREAKQEE	−6.03 (+−2.50)	−3.35	Good	pH 7.15
PGPTVFPAVLY	1.70 (+−1.45)	−5.16	Poor	pH 5.93

Properties such as log⁡*P* (octanol-water coefficient), log⁡*S* (solubility), and isoelectric point determine the oral absorption capability of oligopeptides.

**Table 3:** Shuffled amino acid residues from oligopeptides.

PGKYKLAVSEK	KYKLAPGVSEK, GEKKLVEAPKS, LKYKGPKESVA, YLASEKVKKGP, EKGSAVYGLPK, SPYVEKKAGKL

PGKFYVSSGATTFPAVYL	GPKFVYSGSTATPAFYVL, FGKPSVYSTAGTPAFVYL, SSGAYVTTFPKFAVYGLP, PPGVAKYLVSSGATTFFY, SVYGASVAPKYPLGTFTF, TTFPKFAAYVPLGYVGSS

QVGFSSTAMWNKTFDVSGATQ	WNKTAMTFDFSSVSGQVGATQ, NKATATGVQGMTDFGSVSSQW, MTFDSGSVSWQGQVGTATANK, MTNKATFDATSGGVSVQGSWQ, QVGQTAFSSGSVTAMWKTFDN, SGATQKTDFVQVGFSSNWMAT

QTAMRMIFTALTSPS	MIFTALTAMRSPSQT, TAMRPSSTALQTMIF, FMIQTLTSASPRAMT, MIFQLTTSSAPARTM, SSTTPALQRAFIMMT, TSMSMTITFPAALQR

SGVQPDAPSNDSKDT	VGQPADPNSDSKDTS, PDAPSQNDGKDSSTV, QVPGSAPSDNDSKTD, DKGVSDQPSNDAPST, PSANDDPSQKVDTGS, SNDSGVSDTQPDTAP

PSTLSVSSGTVYEKHEGLS	VSSGTEKHEGLSPSTLSYV, KEHGLSSPLTVSGSSTVYE, GSVSSTLPSLGEHKEVYTS, SVSSLTPSKGEHLSGVYTE, YHTGSSVSLTSPSLGEVKE, SSSTPELHSGEYTGVSKVL

KREAKQEE	AEKREKQE, QEKRAEKE, EEKREKQA, EKQEREAK, KKREAQEE, AKREEEQK, EKEQKRAE, KEAKEREQ

PGPTVFPAVLY	PPVAYLTPFVG, LPTPFGVYVAP, FVPTGVYLPPA, AVPTGYPFVLP, PTFVGYLVAPP, GTYPFVAVLPP

All oligopeptides were shuffled and possible residues were generated randomly.
