# Supplementary material for: In Silico Approach towards Designing Virtual Oligopeptides for HRSV
Source: ScientificWorldJournal. 2014 Nov 27;2014:613293. doi: 10.1155/2014/613293 (PMC4265542; doi:10.1155/2014/613293)
Supplement: Supplementary file 1 — Designed oligopeptides are differentially coloured compared with the model oligopeptide and represented at the right side of the graph which helps in identifying the most stable oligopeptide in terms of their potential energy. [file 613293.f1.pdf]

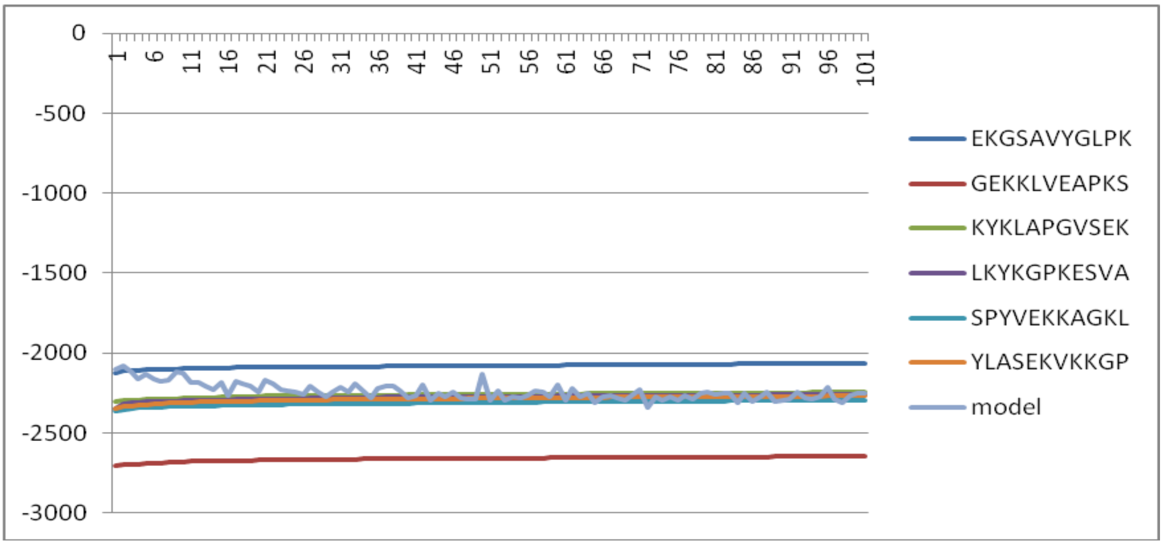

0
-500
-1000
-1500
-2000
-2500
-3000
1
6
11
16
21
26
31
36
41
46
51
56
61
66
71
76
81
86
91
96
101
EKGSAVYGLPK
GEKKLVEAPKS
KYKLAPGVSEK
LKYKGPKESVA
SPYVEKKAGKL
YLASEKVKKGP
model

(A)

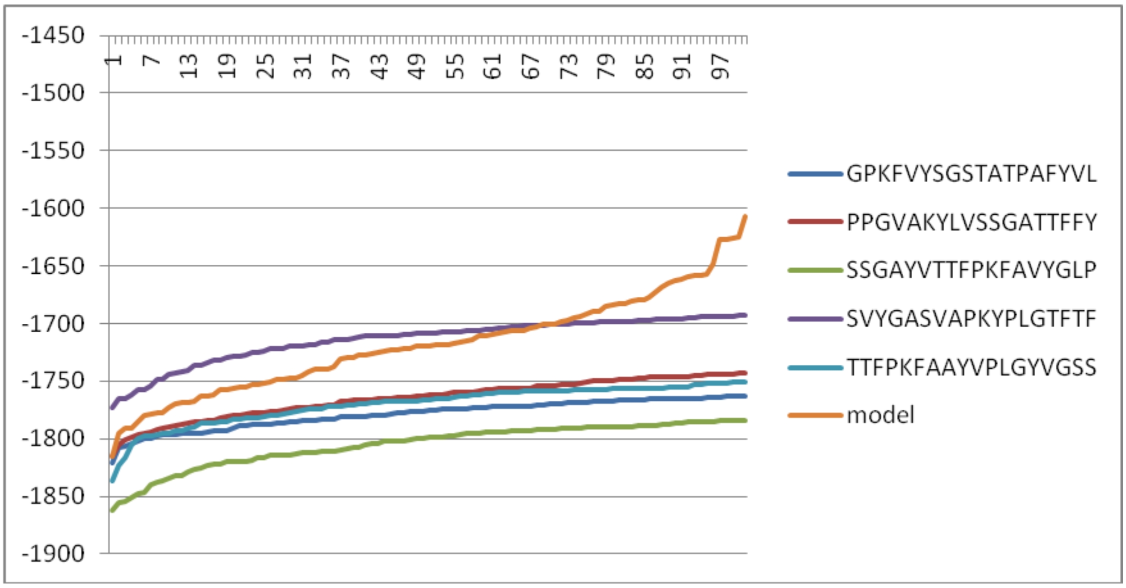

-1450
-1500
-1550
-1600
-1650
-1700
-1750
-1800
-1850
-1900
1
7
13
19
25
31
37
43
49
55
61
67
73
79
85
91
97
GPKFVYSGSTATPAFYVL
PPGVAKYLVSSGATTFFY
SSGAYVTTFPKFAVYGLP
SVYGASVAPKYPLGTFTF
TTFPKFAAYVPLGYVGSS
model

(B)

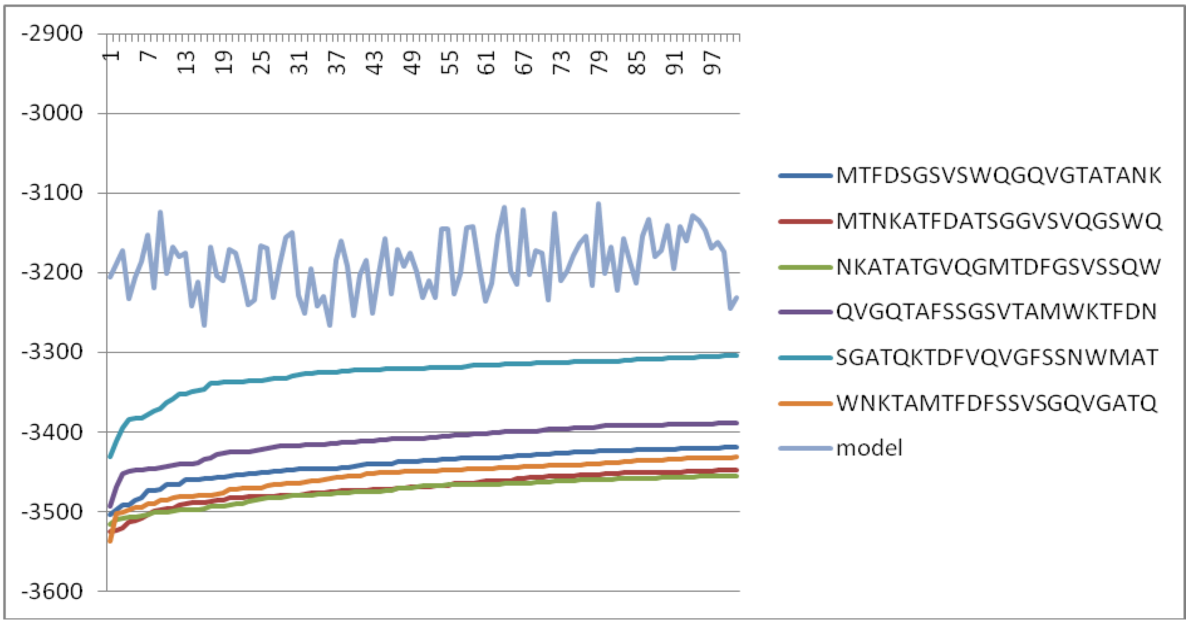
-2900
-3000
-3100
-3200
-3300
-3400
-3500
-3600
1
7
13
19
25
31
37
43
49
55
61
67
73
79
85
91
97
MTFDSGSVSWQGQVGTATANK
MTNKATFDATSGGVSVQGSWQ
NKATATGVQGMTDFGSVSSQW
QVGQTAFSSGSVTAMWKTFDN
SGATQKTDFVQVGFSSNWMAT
WNKTAMTFDFSSVSGQVGATQ
model

(C)

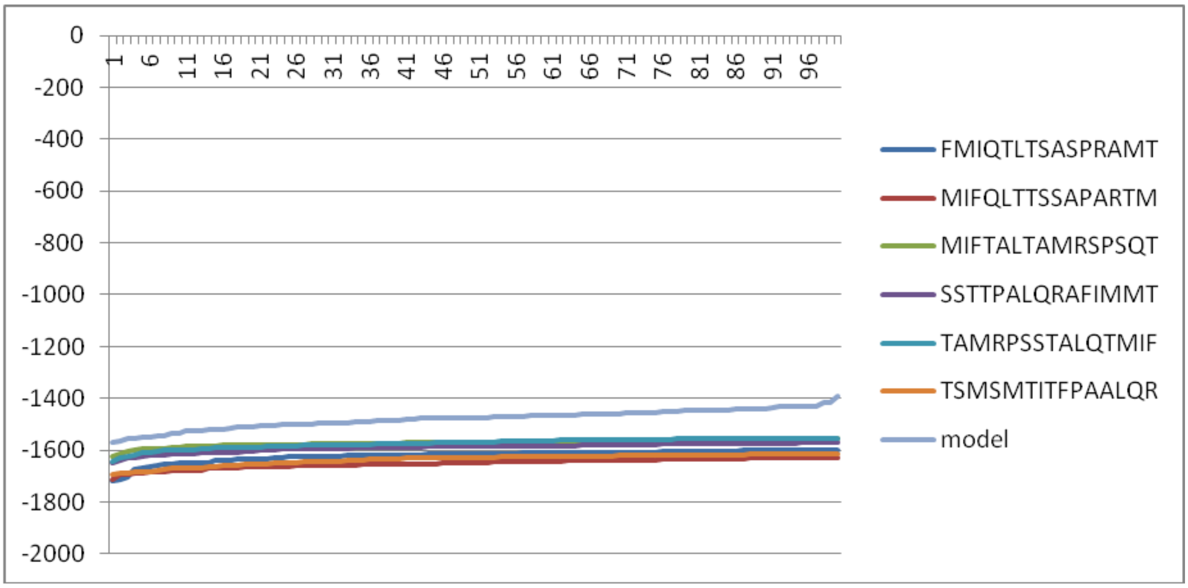
0
-200
-400
-600
-800
-1000
-1200
-1400
-1600
-1800
-2000
1
6
11
16
21
26
31
36
41
46
51
56
61
66
71
76
81
86
91
96
FMIQTLTSASPRAMT
MIFQLTTSSAPARTM
MIFTALTAMRSPSQT
SSTTPALQRAFIMMT
TAMRPSSTALQTMIF
TSMSMTITFPAALQR
model

(D)

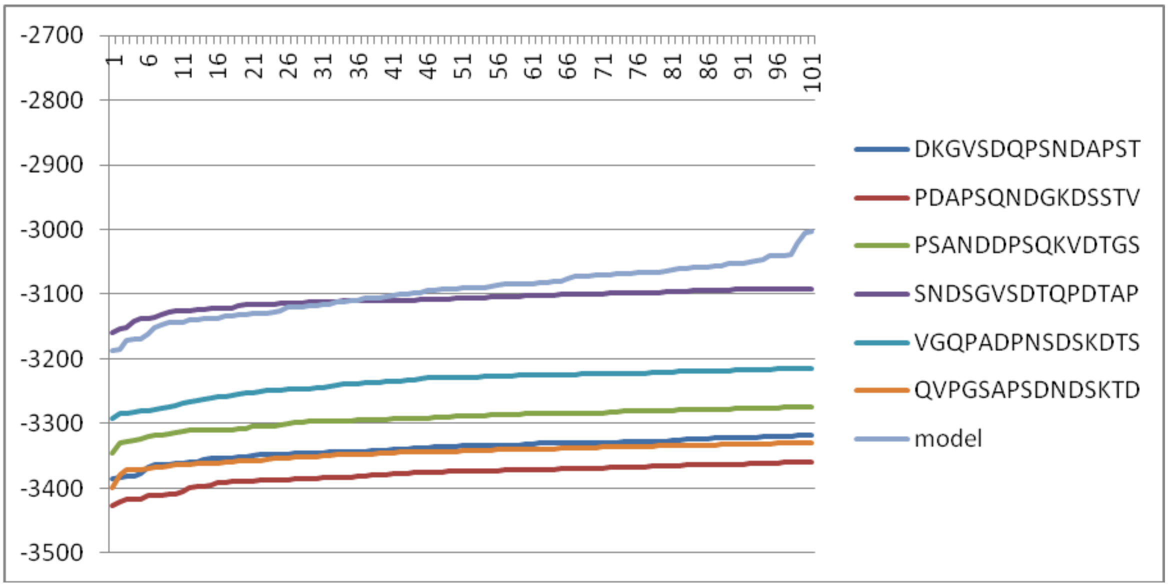
-2700
-2800
-2900
-3000
-3100
-3200
-3300
-3400
-3500
1
6
11
16
21
26
31
36
41
46
51
56
61
66
71
76
81
86
91
96
101
DKGVSDQPSNDAPST
PDAPSQNDGKDSSTV
PSANDDPSQKVDTGS
SNDSGVSDTQPDTAP
VGQPADPNSDSKDTS
QVPGSAPSDNDSKTD
model

(E)

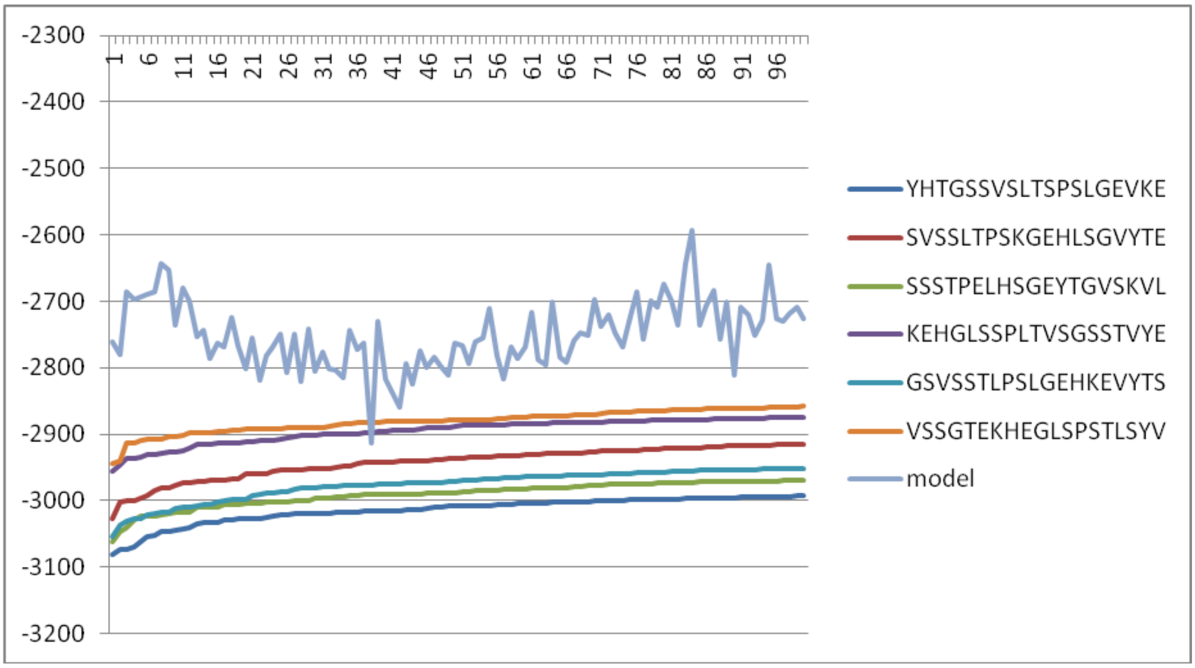
-2300
-2400
-2500
-2600
-2700
-2800
-2900
-3000
-3100
-3200
1
6
11
16
21
26
31
36
41
46
51
56
61
66
71
76
81
86
91
96
YHTGSSVSLTSPSLGEVKE
SVSSLTPSKGEHLSGVYTE
SSSTPELHSGEYTGVSKVL
KEHGLSSPLTVSGSSTVYE
GSVSSTLPSLGEHKEVYTS
VSSGTEKHEGLSPSTLSYV
model

(F)

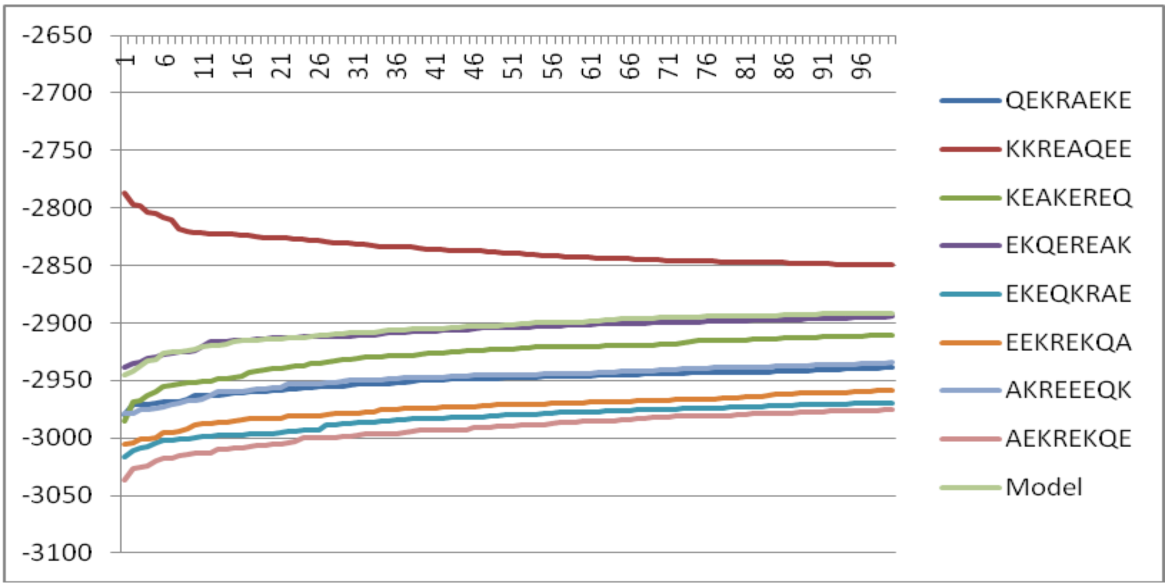

(G)

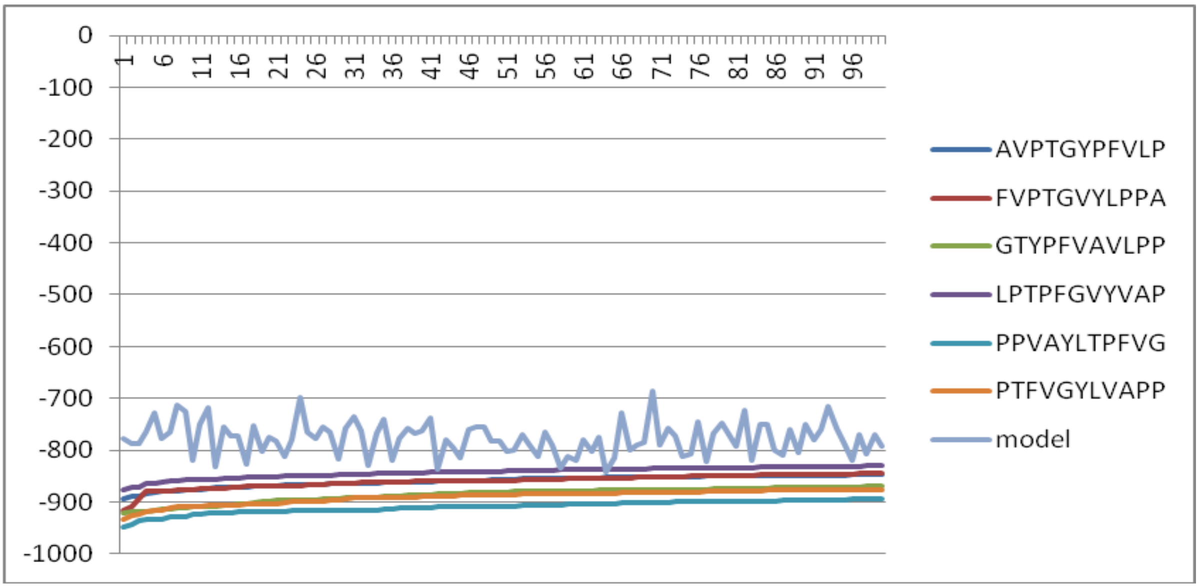

(H)

Graphical representation of potential energies of designed oligopeptides and original oligopeptide represented as model in each graph

(a) **PGKYKLAVSEK (b) PGKFYVSSGATTFPAVYL**
(c)QVGFSSTAMWNKTFDVSGATQ (d) QTAMRMIFTALTSPS
(e) SGVQPDAPSNDSKDT (f) PSTLSVSSGTVYEKHEGLS
(g) KREAKQEE (h) PGPTVFPAVLY

Peptides are differentially coloured and represented at the right side of the graph which helps in identifying the most stable oligopeptide in terms of their potential energy.
